# Increased S-Nitrosylation and Proteasomal Degradation of Caspase-3 during Infection Contribute to the Persistence of Adherent Invasive *Escherichia coli* (AIEC) in Immune Cells

**DOI:** 10.1371/journal.pone.0068386

**Published:** 2013-07-04

**Authors:** Karl A. Dunne, Amr Allam, Anne McIntosh, Stephanie A. Houston, Vuk Cerovic, Carl S. Goodyear, Andrew J. Roe, Scott A. Beatson, Simon W. Milling, Daniel Walker, Daniel M. Wall

**Affiliations:** 1 Institute of Infection, Immunity and Inflammation, University of Glasgow, Glasgow, United Kingdom; 2 School of Chemistry and Molecular Biosciences and Australian Infectious Diseases Research Centre, University of Queensland, St. Lucia, Queensland, Australia; Indian Institute of Science, India

## Abstract

Adherent invasive *Escherichia coli* (AIEC) have been implicated as a causative agent of Crohn’s disease (CD) due to their isolation from the intestines of CD sufferers and their ability to persist in macrophages inducing granulomas. The rapid intracellular multiplication of AIEC sets it apart from other enteric pathogens such as *Salmonella* Typhimurium which after limited replication induce programmed cell death (PCD). Understanding the response of infected cells to the increased AIEC bacterial load and associated metabolic stress may offer insights into AIEC pathogenesis and its association with CD. Here we show that AIEC persistence within macrophages and dendritic cells is facilitated by increased proteasomal degradation of caspase-3. In addition S-nitrosylation of pro- and active forms of caspase-3, which can inhibit the enzymes activity, is increased in AIEC infected macrophages. This S-nitrosylated caspase-3 was seen to accumulate upon inhibition of the proteasome indicating an additional role for S-nitrosylation in inducing caspase-3 degradation in a manner independent of ubiquitination. In addition to the autophagic genetic defects that are linked to CD, this delay in apoptosis mediated in AIEC infected cells through increased degradation of caspase-3, may be an essential factor in its prolonged persistence in CD patients.

## Introduction

Adherent invasive *Escherichia coli* (AIEC) is a Crohn’s Disease (CD) associated pathogen that numerous studies have implicated as a causative agent or contributory factor in disease pathology [1], [2], [3], [4]. AIEC are six times more likely to be isolated from ileal samples of CD patients compared to controls and in intestinal lesions from CD patients *E. coli* strains represented from 50 to 100% of the total bacteria present [1], [5]. Further to this, AIEC isolated from lesions of CD patients are capable of invading epithelial cells and resisting phagocytosis [6]. While the idea of a single bacterial causative agent in CD remains controversial, the majority of host genetic mutations implicated in the disease are linked to autophagy, a programmed cell death (PCD) pathway intrinsically linked to the removal of intracellular bacterial pathogens [7], [8], [9]. In total over 70 host genes, and counting, have been linked to CD susceptibility, pointing to the importance of innate immunity and autophagy as key factors in CD development. Therefore a diminished ability of infected cells to sense an intracellular microbe is no doubt a key feature in CD but the mechanism of AIEC mediated persistence remains uncharacterized.

Unlike closely related species such as *Shigella* and *Salmonella*, and even other pathogenic *E. coli*, AIEC and its virulence factors remain poorly understood. Genome sequencing of LF82, the AIEC type strain isolated from a lesion from a CD patient, indicated that AIEC was most closely related to *E. coli* strains that cause extra-intestinal infections such as meningitis and urinary tract infections and also avian disease causing strains [10]. Despite this, only 4 of 63 of these related *E. coli* strains shared the distinct adherent invasive phenotype [10], [11], [12]. The uniqueness of AIEC was further reinforced by the finding that 115 coding sequences (CDS), or 2.6% of the genome are not found in any other *E. coli* strain and 15 unique CDS not present in pathogenic prokaryotes were also noted [10].

Once AIEC crosses the intestinal epithelium it comes into contact with macrophages, a cell type in which it has been shown to replicate extensively within the acidified phago-lysosome [13], [14]. AIEC in this environment has been reported to multiply an unprecedented 70-fold within the first 48 hours of infection [13]. This potent ability of AIEC to replicate extensively in macrophages while delaying PCD may be a critical factor in the induction of unrestrained, chronic inflammation. While *S*. Typhimurium inhibits apoptotic pathways through up-regulation of host Akt activity through its phosphorylation by the secreted effector SopB, this only allows only temporary inhibition of PCD and any significant increases in bacterial cell number rapidly induced apoptosis [15], [16]. AIEC has no known effector with a corresponding activity to that of SopB and the means by which PCD is delayed in AIEC infected cells remains a mystery.

Programmed cell death as a response to bacterial infection has been well defined with the roles of various PCD pathways in the clearance of infection described in detail [17]. Numerous pathogens however are known to successfully circumvent these pathways, enabling them to establish persistent and sometimes debilitating infections in the host. In the case of circulating immune cells such as macrophages and dendritic cells (DCs) any increase in lifespan of these cells can have detrimental effects on host health with persistent immune cells leading to increases in inflammation and tissue damage and the potential for development of autoimmune diseases [18], [19], [20]. DCs play an important role in CD with specific subsets contributing through the release of high concentrations of pro- and anti-inflammatory cytokines during disease [21]. Induction of the adaptive response by DCs containing AIEC has the potential to exacerbate infection and by disturbing the delicate immune homeostasis in the intestine reduce tolerance to commensal bacteria. AIEC have already been shown to replicate rapidly in intestinal macrophages without inducing apoptosis and the possibility that this is also occurring in DCs cannot be discounted [13].

Ubiquitination is known to be important in the host turnover of apoptotic enzymes such as caspase-3 and caspase-7 with their tagging by ubiquitin trafficking the enzymes for subsequent proteasomal degradation [22], [23]. Bacterial pathogens have been implicated in the targeting of proteasomal recycling pathways with the ubiquitination pathway in particular undermined through the production of bacterial mimics of host E3 ubiquitin ligases [24], [25]. Turnover of caspases within host cells maintains the basal enzyme levels below a certain threshold as a means of reducing or controlling their activity and preventing inadvertent apoptosis. Modifications such as S-nitrosylation can exert similar anti-apoptotic effects by targeting the executioner caspase, caspase-3, blocking its active site through the insertion of an S-nitrosyl group, or priming it for subsequent ubiquitination and proteasomal degradation [26], [27]. The ability of bacterial pathogens to utilize S-nitrosylation in the regulation of their own proteins, or mimics of host enzymes such as ubiquitin ligases, makes these host pathways potential targets of bacterial virulence strategies [28].

Here we describe how during AIEC infection of macrophages and DCs high levels of caspase-3 are trafficked to the proteasome for degradation. At early time points proteasomal degradation of caspase-3 was also seen in uninfected and commensal treated cells indicating this may be a host mechanism of apoptosis control exploited by AIEC. In addition, in macrophages infected with AIEC high levels of S-nitrosylated pro- and active-caspase-3 were observed and these modified forms accumulated after proteasome inhibition in a ubiquitination independent manner. This is the first description of the role of caspase-3 degradation in intracellular survival of a bacterial pathogen and also the first account of proteasomal degradation of S-nitrosylated active caspase-3. In addition to the autophagic defects associated with CD this inhibition of apoptosis by AIEC may be crucial to its persistence.

## Materials and Methods

### Bacterial Strains and Growth Conditions

Strains for infection were back-diluted after overnight growth into 10 ml cultures of RPMI-1640 (Sigma) supplemented with 3% Fetal Calf Serum (FCS) and L-glutamate. These were then grown at 37°C in a shaking incubator at 200 rpm to an optical density of 0.6 before further dilution to give a final multiplicity of infection (MOI) of 100. Strains used for infection were *Salmonella enterica* serovar Typhimurium SL1344 [29], AIEC strain LF82 [1] and *E. coli* F18, a commensal strain isolated from a human intestine [30].

### Cell Culture and Maintenance

RAW 264.7 macrophages obtained from the European Collection of Cell Cultures (ECACC) were maintained in RPMI-1640 media (Sigma) supplemented with 10% FCS (Gibco), L-glutamate and penicillin/streptomycin (both Sigma). The T84 human intestinal epithelial cell line obtained from the American Type Culture Collection (ATCC) was maintained in a 50∶50 mixture of Dulbecco’s Modified Eagles Medium and Hams F-12 Medium (both Sigma) supplemented with 10% FCS, L-glutamate, 15 mM HEPES buffer (pH 7.5), 14 mM sodium bicarbonate and penicillin/streptomycin (Sigma-Aldrich). Cells were maintained at 37°C and 5% CO_2_ with regular media changes. RAW 264.7 macrophages were passaged before reaching 90% confluency while T84 intestinal epithelial cells were passaged at 70% confluency.

### Isolation and Culture of Bone Marrow Derived Dendritic Cells (BMDCs)

Bone marrow derived dendritic cells (BMDCs) were generated using the Flt3 ligand as previously described [31], [32]. Approval for these procedures was given prior to their initiation by the University of Glasgow ethics committee and by the U.K. Home Office under License No. 60/4128. BMDCs were grown in RPMI-1640 supplemented with 10% FCS, 10% Flt3 ligand, L-glutamate and 50 µM ß-mercaptoethanol. After 7 days cells were harvested, stained with fluorochrome conjugated monoclonal antibodies to mouse antigens CD11c (3.9) and B220 (RA3-6B2) (Biolegend) and samples were acquired on an LSR II (BD Biosciences). Classical DCs (cDCs) compromised approximately 30% of total DC cultures and plasmacytoid DCs (pDCs) approximately 31% (Fig. S1).

### Infection of Cells

RAW 264.7 macrophages were seeded at 1×10^5^ cells per well of a 24-well plate 48 hours prior to infection while BMDCs were seeded at 1×10^6^ cells per well of a 24-well plate 24 hours prior to infection. After 24 hours macrophages were treated with 100 ng/ml lipopolysaccharide to induce an activated state. Infections for both cell types were carried out in RPMI media supplemented with 3% FCS and L-glutamate. Infections were carried out at an MOI of 100. After 1 hour the bacteria that had not been internalized were killed by adding 50 ng/ml gentamycin sulfate (Sigma-Aldrich) and the infection allowed proceed. T84 epithelial cells were seeded at 1×10^5^ cells per well 7 days prior to infection. DMEM/Hams F-12 Media was changed at days 1 and 4 and a 100% confluent polarized monolayer of cells allowed form. Infection was carried out at an MOI of 100 and after 2 hours bacteria that had not invaded were killed by the addition of gentamycin sulfate (50 ng/ml). Where stated MG132 (Sigma) an inhibitor of the eukaryotic proteasome was added at a concentration of 10 µM while PYR-41 (Sigma) an inhibitor of eukaryotic E1 ubiquitin enzymes was used at a concentration of 50 µM. The inducible nitric oxide synthase (iNOS) inhibitor N-Nitro-L-arginine methyl ester (L-NAME) (Sigma) was added to RAW 267.4 cells at a concentration of 100 µM where indicated. For long-term infection experiments media was changed every 48 hours.

### Enzyme Activity Assays Post-infection

Cell lysates were prepared post-infection by removing supernatants and scraping off cells in lysis buffer consisting of 2% Triton X-100 in phosphate buffered saline (PBS) at pH 7.5. In addition T84 cell lysates were passed 3 times through a 22-gauge syringe as previously described [30]. Protease inhibitor (Complete Mini, Roche) was added to PBS except where caspase-3 activity was monitored as this inhibited caspase-3 protease activity. Both supernatants and lysates were stored at −20°C until needed. Lactate dehydrogenase (LDH) activity as a measure of cytoxicity was measured in cell supernatants according the manufacturer’s protocol (LDH Cytotoxicity Assay Kit, Abcam). For percentage cytoxicity measurements PBS with 2% Triton-X-100 was as used a positive control to lyse cells (100% cytotoxicity) while the supernatant from uninfected cells was used as a low control (0% cytotoxicity). LDH activity was expressed as a percentage of control wells. In addition a Live/Dead Viability/Cytotoxicity kit (Invitrogen) was used to examine levels of total cell death. The kit was used according to the manufacturer’s protocol. Caspase-3 activity in cell lysates was measured using the Apo-One Homogenous Caspase-3 Activity Kit (Promega). Post-measurement caspase-3 activity was corrected for protein concentration (BCA Protein Assay Kit, Pierce) and expressed as caspase-3 activity Fluorescence Focus Units (FFU) per mg of protein. All enzyme activities were measured using a FluoStar Optima fluorescent plate reader (BMG Biotech).

### Concentration of Ubiquitin Tagged Proteins and Western Blotting

Ubiquitin tagged proteins were concentrated post-infection by passing lysates prepared in PBS, supplemented with 2% Triton-X-100 and protease inhibitor (Complete-Mini, Roche), through a ubiquitin pull-down column (Ubiquitin Enrichment Kit, Pierce) as per the manufacturer’s instructions. During infection of cells to enrich for proteins tagged with ubiquitin or ubiquitin like proteins (UBLs) the proteasome inhibitor MG132 (Sigma) was added at a concentration of 10 µM after invasion and gentamycin treatment and maintained until harvesting of the cell lysates at the end of infection as before. MG132 was used throughout this work for a maximum of 6 hours to reduce any risk of cytotoxicity associated with proteasomal inhibition. The lack of cytotoxicity over this time was confirmed by LDH and Live/Dead Assays. For Western blotting samples were separated through an 8–16% gradient Tris-HCl Gel (Novex Gels, Invitrogen) by polyacrylamide electrophoresis (SDS-PAGE) and transferred to nitrocellulose. Immunoblots were performed using a 1∶1000 dilution of antibodies against caspase-3 and activated-caspase-3 (Cell Signalling Technology). Goat anti-rabbit and goat anti-mouse IgG labeled with horseradish peroxidase (Santa Cruz Biotechnology, Santa Cruz, CA) diluted 1∶2000 were used to detect bands and visualized by enhanced chemiluminescence using the Super Signal ECL kit (Pierce, Rockford, IL).

### Bioinformatic Search for E3 Ubiquitin Ligase Mimics in AIEC

All the CDSs in the genome of *E. coli* LF82 (NC_011993) and its plasmid (CU638872) were collected using Artemis. The CDSs were then uploaded to EffectorFAM, which is a database of Hidden Markov Models for identifying effector proteins designed within the Beatson group (University of Queensland). Since some effectors such as SspH2 from *Salmonella* and YopM from *Yesinia* act as ubiquitin ligase mimics EffectorFAM includes models for predicting these proteins.

In addition the genome of LF82 was searched for the ubiquitin ligase motif (CEDR) that has been identified in all bacterial ubiquitin ligase mimics [33]. Pfam models that are based on the conserved ubiquitin ligase domains were downloaded and used to search the complete set of LF82 protein sequences. The Pfam models that were used; Pfam ID – Description, PF09046 - AvrPtoB E3 ubiquitin ligase; PF11547 - E3 ubiquitin ligase EDD; PF13764 - E3 ubiquitin protein ligase UBR4; PF12483 - GIDE (E3 ubiquitin ligase); PF08647 - BRE1 (E3 ubiquitin ligase); PF00240 - ubiquitin. Next a character search for the ubiquitin ligase motif (CEDR) was performed using Artemis (Genome Research) but no significant matches could be found [34].

A final approach was to use the search tool tblastn (NCBI) to compare the E3 ubiquitin ligase sequences against the nucleotide sequence of the LF82 genome and plasmid [35]. The tool tblastn compares a protein sequence to a nucleotide sequence by translating the nucleotide sequences in all six frames. This method allowed for the possibility that some CDSs were not predicted when the genome was submitted.

### Immunoprecipitation of S-nitrosylated Protein

Nitrosylated protein was labeled through an S–Nitrosylated Protein Detection Assay Kit (Cayman Chemical Company). The manufacturer’s protocol was modified to allow the immunoprecipitation of nitrosylated protein using streptavidin beads as previously described [36]. Briefly, proteins were precipitated after labeling through the use of 100% acetone and resuspended in 0.5 ml of 25 mM Hepes, 1 mM EDTA, 1% sodium dodecyl sulphate (SDS). For streptavidin pull-down, 50 µg of protein from each sample was diluted with 750 µl of neutralization buffer (25 mM Hepes, 100 mM NaCl, 1 mM EDTA, 0.5% Triton X-100, pH 7.7). This solution was tumbled overnight at 4°C with 30 µl of streptavidin agarose beads (Vector Laboratories), which had been pre-washed with neutralization buffer. The beads were pelleted at 200×g for 30 seconds, washed twice with neutralization buffer containing 600 mM NaCl, pelleted and resuspended in SDS–PAGE sample loading buffer. Supernatants of boiled samples were separated by SDS–PAGE and immunoblotted for pro- and active forms of caspase-3 as before.

### Cytokine Assays

BMDCs and RAW 264.7 macrophages were maintained and infected as described. At various time points post-infection supernatants were collected and assayed for the p40 subunit (IL-12, IL-23) or IL-10 production by cytokine ELISA kits (BioLegend), according to the manufacturer’s instructions.

### Detection of Nitric Oxide Levels

Nitric oxide levels were detected using the Griess Assay as previously described but without use of the nitric oxide radical scavenger PTIO after infection [37]. The reaction was read using a FluoStar Optima microplate reader (BMG Biotech).

### Statistical Analysis

All experimental results are expressed as the mean ± standard deviation of an individual experiment done in triplicate. *P* values were calculated according to Student’s *t* test, and values <0.05 were considered statistically significant.

### Ethics Statement

All animal work was performed in strict accordance with the requirements and guidelines of the Animals (Scientific Procedures) Act 1986 outlining the humane and ethical treatment of laboratory animals. Approval for these procedures was given prior to their initiation by the University of Glasgow ethics committee and by the U.K. Home Office (License No. 60/4128).

## Results

AIEC have been reported to rapidly multiply in macrophage-like cells suggesting that AIEC may actively inhibit activation of PCD [13]. To test this further, we examined the role of PCD in AIEC intracellular survival and replication in RAW 264.7 macrophages and murine bone marrow derived dendritic cells (BMDCs). We hypothesized that during AIEC infection PCD is actively inhibited allowing extended periods of AIEC intracellular growth.

**Figure 1:**
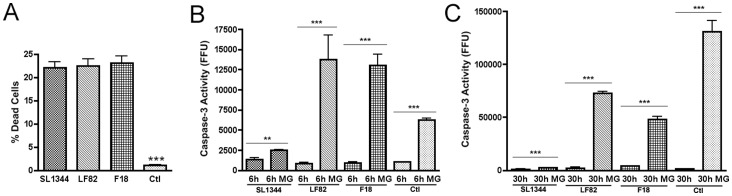
Caspase-3 accumulates in LF82 infected RAW 264.7 cells after proteasome inhibition. (A) Cell death of RAW 264.7 cells at 6 hours post infection (hpi). The percentage of dead cells were calculated using a LIVE/DEAD stain and expressed as a percentage of the total number of cells present per well of a 24 well plate. (B) Accumulation of caspase-3 activity in RAW 264.7 cells after proteasome inhibition from 0–6 hpi. Caspase-3 activity in RAW 264.7 cells was measured at 6 hpi and expressed as activity, measured in fluorescence focus units (FFU) per mg of protein. Measurements were carried out in the presence or absence of 10 µM MG132. (C) Accumulation of caspase-3 activity in RAW 264.7 cells after proteasome inhibition from 24–30 hpi. Caspase-3 activity in RAW 264.7 cells was measured at 30 hpi and expressed as activity, measured in fluorescence focus units (FFU), per mg of protein. Measurements were carried out in the presence or absence of 10 µM MG132. All experiments were repeated at least three times in triplicate and data from representative experiments are shown. Data was analyzed by an unpaired Student’s *t*-test. Statistically significant relationships are denoted. *P* values **<0.05, ***<0.005.

To understand in greater detail the host response to infection, both RAW 264.7 cells and BMDCs were initially infected for 6 hours. This time point allowed us to study infection whilst also avoiding any potential longer term cytotoxic side effects associated with inhibiting either ubiquitination or proteasome activity. At 6 hours post infection (hpi) Live/Dead staining of the infected RAW 264.7 cells indicated no significant difference between samples infected with *S*. Typhimurium, *E. coli* F18 or *E. coli* LF82 in progression towards PCD (Fig. 1A). *E. coli* F18 is a commensal non-pathogenic strain isolated from a human intestine while LF82 is the type strain for AIEC that was isolated from a CD patient [30], [38]. Similarly, caspase-3 activity used as an indicator of the progression of RAW 264.7 cells toward apoptosis indicated no difference at 6 hpi between uninfected and infected cell types (Fig. 1B). To determine if degradation of caspase-3 via the proteasome was masking increased levels of caspase-3 activity we used MG132 (10 µM) to inhibit proteasomal activity allowing the accumulation of proteins trafficked to the proteasome. Upon addition of MG132 a 16.3-fold increase in caspase-3 activity was observed in RAW 264.7 cells treated with the AIEC type strain LF82 and to a lesser extent commensal *E. coli* F18 (14.3-fold) and uninfected cells (6.0-fold). This indicates that caspase-3 is degraded through the proteasome at high levels in AIEC and F18 infected RAW 264.7 cells, but not in those infected with SL1344 (1.8-fold).

In order to study this phenomenon at a later time point in infection, MG132 was added between 24 and 30 hpi. After inhibition of the proteasome a 32-fold increase in caspase-3 activity was seen in LF82 infected cells (Fig. 1C). In F18 treated cells caspase-3 activity was lower (12-fold) but in uninfected cells the levels of activity increased approximately 90-fold. Again there was little increase in accumulated caspase-3 activity in the SL1344 infected cells (2.4-fold) although at this point the bacterial numbers recovered from RAW 264.7 cells were comparable for SL1344 {8.0 (±6.0)×10^6^} and LF82 {5.56 (±3.71)×10^6^} while no F18 were recovered. The further increase in caspase-3 degradation between 24–30 hpi noted in LF82 infected RAW 264.7 cells was unexpected given the level of intracellular bacteria present and it was in stark contrast to virtually non-existent turnover of caspase-3 in SL1344 infected macrophages.

**Figure 2:**
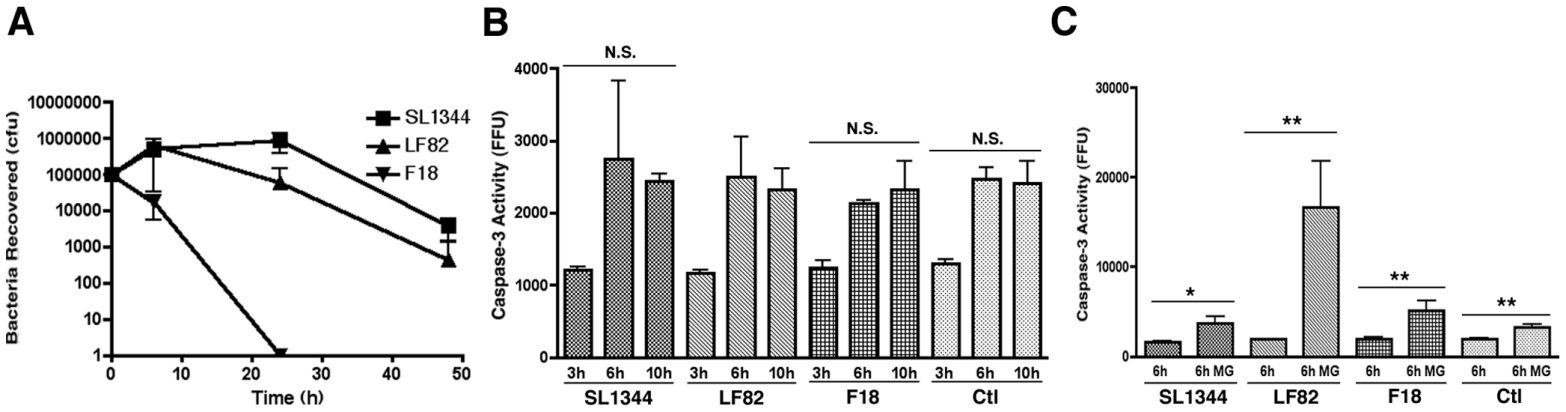
Caspase-3 accumulates post-proteasome inhibition in LF82 BMDCs. (A) Recovery of bacteria post intracellular survival and growth in BMDCs. After infection of BMDCs at an MOI of 100, bacteria were recovered and counted over 48 hpi. Intracellular growth curves were repeated at least three times in triplicate and data from a representative experiment is shown. (B) Caspase-3 activity in BMDCs at 3, 6 and 10 hpi. Caspase-3 activity was measured for the first 10 hpi and expressed as activity per mg of protein recovered. Caspase-3 activity assays were repeated at least three times in triplicate and data from a representative experiment is shown. No significant difference was noted between LF82 infected and any of the other infected or uninfected control cells. (C) Accumulation of caspase-3 activity in BMDCs after proteasome inhibition from 0–6 hpi. Caspase-3 activity in BMDCs was measured at 6 hpi and expressed as activity, measured in fluorescence focus units (FFU) per mg of protein. Measurements were carried out in the presence or absence of 10 µM MG132. All experiments were repeated at least three times in triplicate and data from a representative experiment is shown. Data was analyzed by an unpaired Student’s *t*-test. Statistically significant relationships are denoted. NS = Not significant. *P* values *<0.01, **<0.05.

Pathogenic bacteria are known to infect and replicate within circulating DCs which can then act as a means of spread from the initial focal point of infection [39], [40], [41]. Using BMDCs (Fig. S1) we firstly characterized the ability of LF82 and SL1344 to survive and multiply within these cells with each showing a comparable survival rate intracellularly over 48 hours while non-pathogenic *E. coli* F18 was not recovered after 6 hpi (Fig. 2A). The level of replication of LF82 within BMDCs was again comparable to SL1344 but was not to the levels seen previously in macrophage-like cells [13]. At 3, 6 and 10 hpi caspase-3 activity was not significantly different between infected and uninfected BMDCs (Fig. 2B) and there was no significant difference in the levels of BMDC cytotoxicity induced by the bacteria at 3 hpi or at 6 hpi in the presence or absence of MG132 (Fig. S2). However, as with RAW 264.7 cells, upon blocking of proteasomal turnover with MG132 (10 µM) there was again a dramatic accumulation of caspase-3 activity in LF82 infected cells (8.7-fold) at 6 hpi (Fig. 2C). This was in contrast to BMDCs treated with F18 (2.6-fold) and also the control uninfected BMDCs (1.7-fold). Levels of caspase-3 activity in SL1344 infected cells (2.3-fold) mirrored those seen with the commensal F18 strain. This indicates high levels of caspase-3 are targeted to the proteasome for degradation in LF82 infected BMDCs. Again this is in contrast to BMDCs infected with pathogenic SL1344 where caspase-3 turnover is low. Surprisingly, and unlike in RAW 264.7 cells, in BMDCs caspase-3 turnover is not seen to the same extent in uninfected cells and suggests that different mechanisms of regulating apoptosis may be used in DCs.

**Figure 3:**
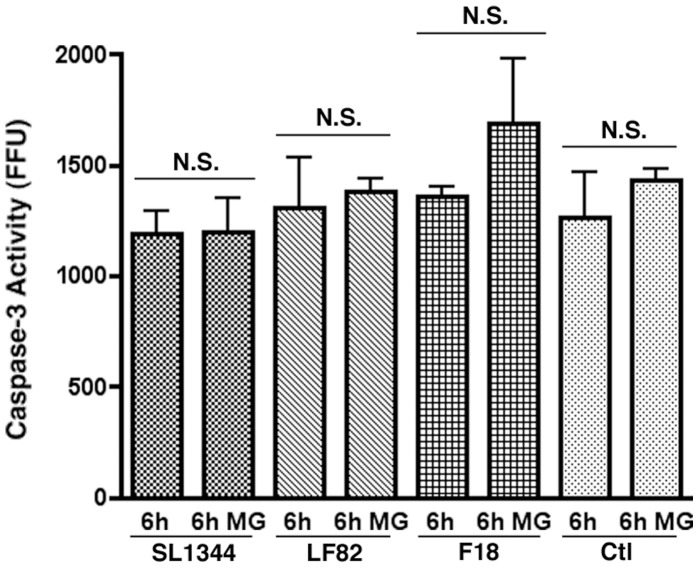
The effect of proteasome inhibition on caspase-3 activity in T84 intestinal epithelial cells from 0–6 hpi. Caspase-3 activity in T84s was measured at 6 hpi and expressed as activity, measured in fluorescence focus units (FFU) per mg of protein. Measurements were carried out in the presence or absence of 10 µM MG132. Experiments were repeated at least three times in triplicate and data from a representative experiment is shown. Data was analyzed by an unpaired Student’s *t*-test. NS = Not significant.

In intestinal epithelial cells we had previously noted the crucial role that caspase-3 plays during initial infection by SL1344 [29]. LF82 are also noted to adhere to and invade these cells causing damage to intestinal epithelial cells and disrupting barrier integrity [42]. To examine the role that caspase-3 turnover may play during infection of the intestinal epithelium we utilized the T84 human intestinal epithelial cell line and again treated with 10 µM MG132 to inhibit proteasome activity (Fig. 3). However, unlike circulating immune cells we detected no increase in caspase-3 activity post-MG132 addition indicating that proteasomal turnover of caspase-3 is not a significant factor in intestinal epithelial cells at early time points during infection or indeed in uninfected cells.

Caspase-3 degradation by the proteasome has been described as a potential means of maintaining caspase-3 levels below the threshold needed for the induction of apoptosis in the cell [23]. This form of protection from apoptosis has been described in uninfected cells and can be mediated by ubiquitination or poly-ubiquitination of caspase-3. Due to the presence of ubiquitin ligase mimics in numerous bacterial strains we speculated that a ubiquitin ligase mimic may also be present in LF82 that can carry out this modification [24], [43]. All the CDSs in the genome of *E. coli* LF82 (NC_011993) and its plasmid (CU638872) were collected using Artemis [34]. The CDSs were then uploaded to EffectorFAM, a database of Hidden Markov Models for identifying effector proteins designed within the Beatson group at the University of Queensland. The genome of LF82 was searched for the known ubiquitin ligase motif (CEDR) and also using Pfam models based on known conserved ubiquitin ligase domains [33]. However, no hits corresponding to ubiquitin ligase mimics in LF82 were found with these models. A final approach confirmed these findings using tblastn to compare the E3 ubiquitin ligase sequences against the nucleotide sequence of the LF82 genome and plasmid [35]. We concluded that it is highly unlikely that the *E. coli* LF82 genome encodes any ubiquitin ligase mimics.

**Figure 4:**
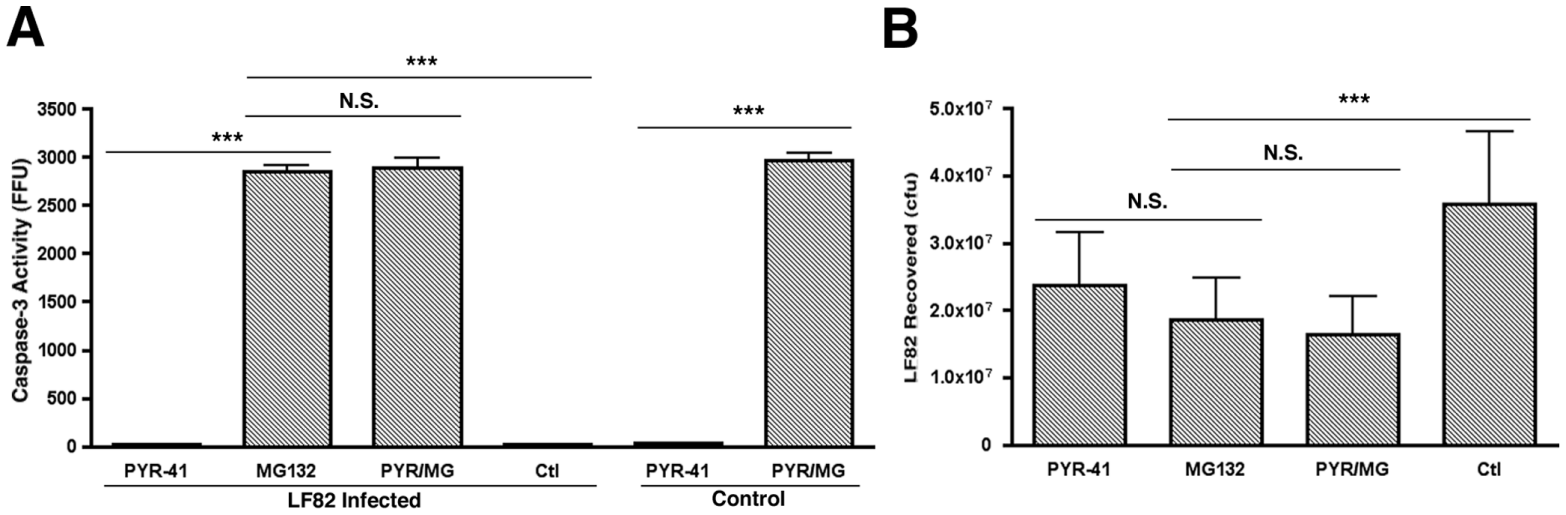
Caspase-3 build up in RAW 264.7 cells post-proteasome inhibition is independent of ubiquitination. (A) The effect of inhibition of E1 ubiquitin activating enzymes on caspase-3 accumulation. RAW 264.7 cells were infected with LF82 or left uninfected (control) and treated for 6 hpi with 10 µM MG132, 50 µM PYR-41 or a combination thereof (PYR/MG). Caspase-3 activity was measured and expressed as activity per mg of protein recovered. Infected but untreated RAW 264.7 cells were used as controls also. Measurements were carried out in the presence or absence of 10 µM MG132. (B) The effect of inhibition of ubiquitination and proteasomal degradation on bacterial intracellular survival. RAW 264.7 cells were infected with LF82 and treated for 6 hpi with 10 µM MG132, 50 µM PYR-41 or a combination thereof (PYR/MG). Bacterial colony counts were then carried out on bacteria recovered from each well and expressed as colony forming units (cfu). Untreated and infected RAW 264.7 cells were used as an additional control. Experiments were repeated at least three times in triplicate and data from a representative experiment is shown. Data was analyzed by an unpaired Student’s *t*-test. Statistically significant relationships are denoted. NS = Not significant. *P* value **<0.05, ***<0.005.

The probable lack of a bacterial ubiquitin ligase mimic however does not discount host mediated ubiquitination of caspase-3 as a possible response to infection by LF82. To investigate this further, ubiquitinated proteins were immunoprecipitated and probed for caspase-3 but no ubiquitinated caspase-3 was observed (data not shown). As the immunoprecipitation assay kit used was designed to identify poly-ubiquitinated proteins we also carried out the reverse immunoprecipitation using anti-caspase-3 antibody and probed for mono- or poly-ubiquitin but again no caspase-3 ubiquitination was observed (data not shown). To conclusively discount the role of ubiquitination in proteasomal degradation of caspase-3 during LF82 infection, RAW 264.7 cells were treated with 50 µM PYR-41. This inhibitor completely blocks the activity of the E1 activating enzyme that catalyzes the first step in ubiquitination. Caspase-3 activity was not increased in the presence of PYR-41 and there was no alteration in caspase-3 accumulation upon addition of MG132 indicating that ubiquitination is not responsible for its targeting to the proteasome during LF82 infection (Fig. 4A). Throughout this work there was no significant difference in bacterial numbers within RAW 264.7 cells treated with PYR-41 or MG132, or a combination of both (Fig. 4B).

**Figure 5:**
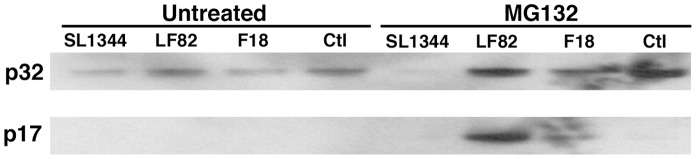
Build-up of S-nitrosylated forms of caspase-3 in RAW 264.7 macrophages when the proteasome is inhibited. S-nitrosylated proteins were probed with anti-caspase-3 antibody to examine accumulation of pro- (p32) and active (p17) forms of caspase-3 after inhibition of proteasomal degradation with 10 µM MG132. Western blotting was repeated three times and a representative Western blot is shown.

Alternative methods of caspase-3 modification were then explored that could explain how it is targeted to the proteasome for degradation. Caspase-3 can also be modified through a process of S-nitrosylation, whereby an S-nitrosyl group is linked to the active site cysteine of caspase-3 (Cys163) thus maintaining the enzyme in an inactive state [26], [27], [44]. Using the biotin-switch technique S-nitrosylated protein generated in the presence or absence of MG132 was biotin labeled and immunoprecipitated before being probed with anti-caspase-3 antibody [36], [45] (Fig. 5). S-nitrosylated caspase-3 was detected in all RAW 264.7 samples at 6 hpi. However with the addition of 10 µM MG132 to inhibit proteasomal degradation there was a dramatic increase in S-nitrosylated inactive pro-caspase-3 (p32) in the LF82, F18 and uninfected samples. In addition, and most significantly, there was a striking accumulation of S-nitrosylated active caspase-3 (p17) in LF82 infected cells upon addition of MG132 while no S-nitrosylated active caspase-3 (p17) was detected in SL1344 infected or uninfected cells (Fig. 5 and S7). This indicates that in macrophages infected with LF82 there is a significant increase in S-nitrosylation of both pro- and active caspase-3 and these accumulate to high levels when the proteasome is inhibited. S-nitrosylation of the active form of caspase-3 has not previously been described in the literature and therefore this unique modification may be a very direct means for persistent bacterial pathogens to delay apoptosis.

**Figure 6:**
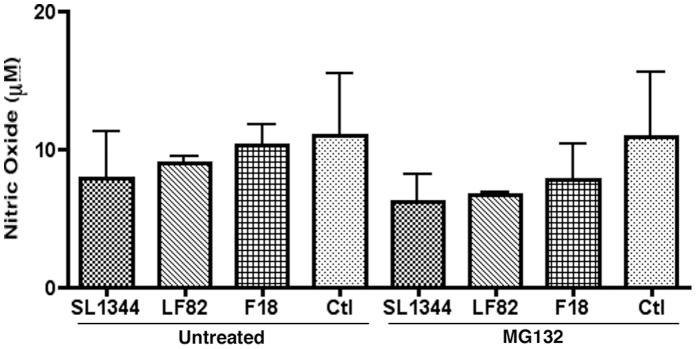
Nitric oxide production in RAW 264.7 cells 6 hpi. NO levels in the supernatants of RAW 264.7 cells were measured using the Griess assay. Levels in cells treated with MG132 (10 µM) were lower than untreated cells but there was no significant difference between any of the uninfected samples or controls. This decrease in NO production upon MG132 addition indicated that the increase in S-nitrosylation of caspase-3 was not due to increased NO production in response to infection. Experiments were repeated at least three times in triplicate and data from a representative experiment is shown.

**Figure 7:**
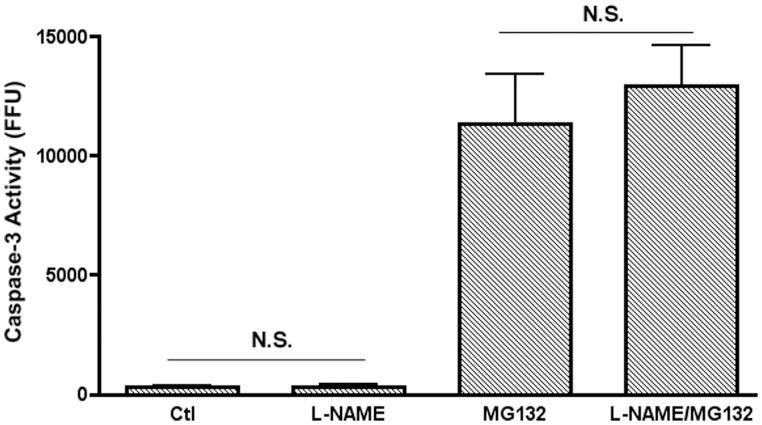
The effect of iNOS inhibition on the observed increase in caspase-3 activity post-proteasomal blocking. RAW 264.7 cells were infected with LF82 and treated with or without the proteasomal inhibitor MG132 (10 µM) as before. Addition of the iNOS inhibitor L-NAME (100 µM) did not cause any decrease in caspase-3 activity in cells where the proteasome was blocked. *P*-value NS (Not significant).

S-nitrosylation levels within eukaryotic cells have been shown to be intrinsically linked to nitric oxide levels the production of which is increased during bacterial infection [46]. Nitric oxide (NO) levels in infected RAW 264.7 cells were studied for any increase upon LF82 infection and to ensure that MG132 treatment over the first 6 hours of infection was not responsible for inducing an increase in NO production. No significant difference in induced NO was seen in infected or control RAW 264.7 cells at 6 hpi with or without 10 µM MG132 treatment (Fig. 6). In addition in order to further ensure that increased NO production over the first six hours of infection was not having an effect on caspase-3 S-nitrosylation and subsequent degradation we inhibited inducible nitric oxide synthase (iNOS) using the iNOS specific inhibitor L-NAME. This inhibition did not reduce the proteasomal dependent accumulation of caspase-3 activity (Fig. 7). This further clarified that the observed effect was not due to increased nitrosative stress in response to LF82 infection of RAW 264.7 macrophages.

**Figure 8:**
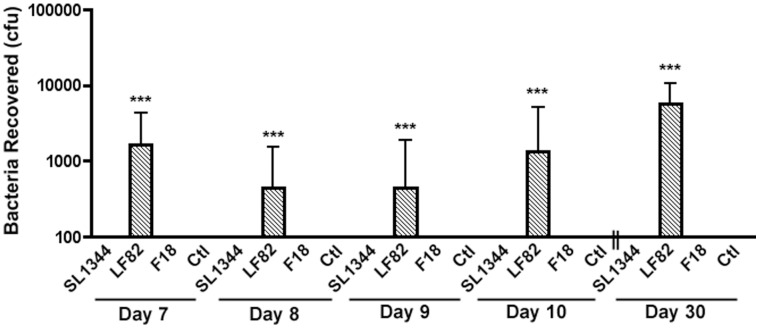
Recovery of bacteria from RAW 264.7 cells up to 30 days post infection. RAW 264.7 cells were infected as described and maintained in 3% FCS for a period of 30 days with regular media changes and maintenance of gentamycin at 50 µg/ml. Intracellular bacteria were harvested as before by lysing RAW 264.7 cells. Experiments were repeated at least three times in triplicate and data from a representative experiment is shown. Data was analyzed by an unpaired Student’s *t*-test. Statistically significant relationships are denoted. *P* value ***<0.005.

We lastly attempted to understand the significance of S-nitrosylation of caspase-3 to long-term infection and inflammation associated with LF82. The numbers of LF82 recovered from RAW 264.7 cells over the first 72 hours of infection mirrored those seen with pathogenic SL1344 but were in contrast to commensal F18 which disappeared rapidly post infection. Interestingly, and perhaps more significantly however, we did see persistence of LF82 in RAW 264.7 cells that were maintained for 30 days post infection (Fig. 8). This was in contrast to SL1344 and *E. coli* F18 infected RAW 264.7 cells where bacteria were not recovered 1 day post infection for F18 and 5 days post infection for SL1344. In addition studies of cytokine levels secreted by RAW 264.7 cells and BMDCs over the first 72 hours of infection indicated that LF82 does not induce an increased inflammatory reaction when compared to commensal *E. coli* F18 and it does not repress the production of the anti-inflammatory cytokine IL-10 (Fig. S3–S6). Together this data indicates that AIEC does not induce an acute inflammatory response in RAW 264.7 cells or BMDCs. Therefore persistence within immune cells mediated by S-nitrosylation of caspase-3 may have an important role, both in inducing longer term persistent inflammation through intracellular survival and also acting as a seeding mechanism for further infection.

## Discussion

AIEC is an atypical opportunistic intestinal pathogen that differs from acute pathogens such as *S*. Typhimurium in its ability to carry out prolonged periods of intracellular replication. This rapid intracellular growth is unprecedented amongst bacterial pathogens and suggests a unique phenomenon whereby infected host cells are maintained in a persistent state unable to induce PCD. Here we show for the first time a unique phenotype for AIEC infected RAW 264.7 cells and BMDCs with increased proteasomal degradation of caspase-3 inhibiting the ability of AIEC infected cells to induce apoptosis. This degradation of caspase-3 was concomitant with increased S-nitrosylation of caspase-3 and independent of ubiquitination which has previously been described as the mediator of such degradation in uninfected cells [23]. In addition to the already established ability of AIEC to prosper in cells where autophagic defects have been noted, this additional observation of the inhibition of apoptosis in AIEC infected immune cells may explain why AIEC in particular, as opposed to other intestinal pathogens, prosper under the conditions noted in CD patients [1], [5].

Bacteria that enter into the mammalian intestine can be divided into three main types; bacteria that grow intracellularly causing acute infection (*S*. Typhimurium), bacteria that grow intracellularly and persist (AIEC, *Mycobacterium* species), and non-pathogenic bacteria that are taken up and killed and are unable to instigate intracellular replication (commensals). The reasons behind AIEC persistence intracellularly have to date remained elusive and cannot be explained by the autophagic defects associated with CD as these were not found in macrophages in which AIEC were initially shown to replicate so effectively [13]. Surprisingly when PCD was analyzed, the host response to AIEC differed dramatically from that of another intestinal pathogen *S*. Typhimurium, with the response to AIEC at early time points mirroring the apoptosis and necrosis levels seen with a non-pathogenic commensal strain of *E. coli*. As proteasomal degradation of caspase-3 has previously been described as a means of preventing caspase-3 build up and averting spontaneous apoptosis in uninfected cells we speculated that similarly, despite rapid intracellular growth, caspase-3 is maintained below threshold levels in AIEC infected cells [22], [23]. Increased turnover of caspase-3 in the *E. coli* treated host cells for the first 6 hpi occurred at a level similar to that seen in uninfected cells and in contrast to *S*. Typhimurium infected cells where caspase-3 turnover was not seen. Caspase-3 turnover in AIEC infected cells further increased at 24 hpi but remained unchanged and at low levels in S. Typhimurium infected cells. As *S*. Typhimurium also grows intracellularly, albeit for a shorter period during acute infection, we concluded that these pathogens employ vastly different strategies to inhibit apoptosis. *S*. Typhimurium inhibition of PCD is dependent on SopB phosphorylation and activation of the apoptosis inhibitor Akt as previously described and not through caspase-3 turnover as is the case with AIEC [47]. Therefore, it is likely that caspase-3 levels are tightly controlled in *S*. Typhimurium infected immune cells, removing the need to later induce its degradation in a manner similar to AIEC. Proteasomal degradation of caspase-3 was also seen in RAW 264.7 cells and BMDCs upon the uptake of commensal bacteria and in uninfected cells implying this is also a strategy employed to prevent spontaneous cell death in the absence of infection. While AIEC intracellular levels increased over the first 6 hpi *E. coli* F18 was completely removed. Yet no significant difference in the rate of proteasomal degradation of caspase-3 between these treated cells was noted in RAW 264.7 cells or BMDCs with these cells apparently unable to distinguish the pathogenic potential of AIEC.

Ubiquitination of caspase-3 is a mechanism utilized by uninfected cells to maintain a basal level of caspase activity within cells thus preventing apoptosis and extending their lifespan [22], [23]. We speculated that mimics of host ubiquitin ligases, the enzymes that carry out this reaction, may be present in AIEC as they have been found in intestinal pathogens including a number of pathogenic *E. coli* strains [24], [25]. However despite in depth screening no ubiquitin ligase mimics were detected in LF82, the type strain of AIEC. In addition complete inhibition of ubiquitination in AIEC infected cells using the chemical inhibitor of E1 activating enzymes had no effect on proteasomal degradation of caspase-3. This is not unprecedented as numerous viral infections induce proteasomal degradation of host proteins in a ubiquitin independent manner although the mechanism or pathway involved has to date remained elusive [40], [48].

Alternative modifications of caspase-3 have been described in the literature with S-nitrosylation of caspase-3 the most interesting given its role in caspase-3 turnover and also its ability to inhibit caspase-3 activity [44], [49]. In addition the recent description of roles for host derived nitrates in facilitating *E. coli* infection of the gut and for nitric oxide during *M. tuberculosis* infection add to the significance of these nitrosyl modifications during persistent infection [50]. During S-nitrosylation an S-nitrosyl group is reversibly attached to the active site cysteine residue (C163) of caspase-3 inhibiting enzymatic activity [26]. Upon addition of the proteasomal inhibitor MG132 to AIEC infected cells, a significant increase was noted in S-nitrosylated caspase-3 protein levels indicating that S-nitrosylation was trafficking caspase-3 to the proteasome. This occurs independently of ubiquitination but whether S-nitrosylation alone is capable of inducing proteasomal degradation is unknown. Previous studies have characterized S-nitrosylation of proteins as acting as a primer for ubiquitination and subsequent proteasomal degradation so it cannot be ruled out that although ubiquitination is not responsible, an alternative pathway may cause subsequent additional modifications to caspase-3 post-nitrosylation [51].

AIEC infected RAW 264.7 cells exhibited a startling turnover of activated, as well as pro-, caspase-3, a phenomenon to our knowledge that has not been described in the literature (Fig. 5). This accumulation of active caspase-3 after proteasome inhibition suggests that AIEC infected cells are capable of converting pro-caspase-3 into its activated form in response to AIEC infection in order to undergo apoptosis. This activated caspase-3 however, which was seen at approximately 10-fold higher levels in AIEC infected cells when the proteasome was blocked, is trafficked for degradation or rendered inactive by S-nitrosylation. Pro-caspase-3 is known to be inhibited by S-nitrosylation of the active site cysteine, preventing any inadvertent activity. However, to our knowledge this is the first description of S-nitrosylation of the active form of caspase-3 and so it is not known if the S-nitrosyl group can attach to the active site cysteine or if modification of the enzyme occurs at an alternative cysteine residue. The effect of S-nitrosylation of active caspase-3 on the enzymes activity is unknown but our data has led us to speculate it has a similar inhibitory effect. While our enzyme activity assays for caspase-3 indicate a high turnover of caspase-3 through proteasomal degradation, in AIEC infected cells there is an additional accumulation of active caspase-3 in the cell that may not be detected due to S-nitrosylation of its active site rendering it inactive. This would explain the far higher levels of active caspase-3 detected by western blotting in AIEC infected RAW 264.7 cells not being fully reflected in caspase-3 enzyme activity assays.

S-nitrosylation of caspase-3 can be host-mediated with numerous studies implicating nitric oxide (NO), X-linked inhibitor of apoptosis (X-IAP) and thioredoxin in the induction of caspase-3 S-nitrosylation [44], [46], [52], [53], [54]. Seven different caspases are inhibited by NO mediated reversible S-nitrosylation and NO, in addition to thioredoxin, is known to cause S-nitrosylation of the critical cysteine residue in the active site of caspase-3 [44], [46]. The mechanism of caspase-3 S-nitrosylation has yet to be elucidated although the production of NO in response to infection by AIEC could induce the inhibition of caspase-3 in an indirect manner. Indeed during viral infection inducible nitric oxide synthase (iNOS) activity was found to have an anti-apoptotic effect and NO increases survival of monocytes by influencing caspase-3 and caspase-9 activation [55], [56]. However, during LF82 infection there was no significant difference in NO production between LF82 infected RAW 264.7 cells and other infected or control cells suggesting a role for NO is unlikely (Fig. 6). In addition, inhibition of iNOS through the use of the iNOS specific inhibitor L-NAME had no effect on the accumulation of caspase-3 activity after the addition of MG132 to inhibit the proteasome (Fig. 7). These results suggest that an increase in intracellular NO levels is not responsible for mediating caspase-3 S-nitrosylation and degradation during acute LF82 infection. As *S*. Typhimurium is known to actively inhibit pathways linked to NO production it may be the case that AIEC also inhibits NO production to promote intracellular survival and this is currently being further examined along with potential roles for host X-IAP and thioredoxins in this process [57].

While S-nitrosylation of caspase-3 we believe is most likely host-mediated the potential role of bacterial virulence factors cannot be discounted. AIEC does not express a type III secretion system (T3SS) but is known to encode a type VI secretion system (T6SS) and is speculated to use outer membrane vesicles (OMVs) as a means of bacterial protein delivery [10], [58]. While classical virulence factors have not been found in the AIEC genome the presence of numerous uncharacterized genes allows for the possibility that unique virulence factors or toxins are being delivered into host cells. The potential overlap in function between S-nitrosylation pathways in bacteria and host cells may also be significant with bacteria, including LF82, expressing proteins that mediate S-nitrosylation of their own bacterial proteins [59], [60]. LF82 in addition encodes two thioredoxins that share homology with mammalian thioredoxins, proteins that are known to S-nitrosylate caspase-3 [10], [44].

While S-nitrosylation could be carried out directly by a bacterial virulence factor an indirect effect through perturbation of host cell pathways appears more plausible. The transfer of S-nitrosyl groups between specific proteins, or transnitrosylation, plays a crucial role in controlling the apoptotic cascade with S-nitrosylation conversely controlling the activation state of both pro- and anti-apoptotic proteins. With the X-linked inhibitor of apoptosis (XIAP) known to transnitrosylate caspase-3, and vice versa, it is possible that inhibition of this XIAP reaction may also be targeted by AIEC in a similar fashion to *Pseudomonas aeruginosa* which stabilizes XIAP and delays apoptosis during infection [61]. Perturbation of such a transnitrosylation reaction would maintain caspase-3 in an inactive state while XIAP, without an S-nitrosyl group, would remain constitutively active meaning apoptosis would be inhibited in two distinct ways.

The outcome of AIEC maintaining immune cells in a persistent state has potential to contribute to and exacerbate intestinal inflammation. Increases in the lifespan of immune cells can be detrimental to intestinal homeostasis through the release of pro-inflammatory cytokines and in some cases is speculated to contribute to the development of autoimmune disorders [18], [19], [20]. Upon examination of cytokine levels released by infected RAW 264.7 cells or BMDCs over the first 72 hours of infection *E. coli* treated cells secreted more pro-inflammatory p40 (IL-12/23) than SL1344 infected cells. However there was no significant difference between levels of p40 (IL-12/23) or anti-inflammatory IL-10 released by RAW 264.7 cells or BMDCs treated with LF82 or non-pathogenic F18 (Fig. S3–S6). The role of these cytokines in longer term infections is currently being further examined. However we could recover AIEC up to 30 days post-infection of RAW 264.7 cells at levels of approximately {5.67 (±4.97)×10^4^} per well (Fig. 8). In contrast no SL1344 or F18 were recovered after 5 days or 1 day of infection respectively. Therefore the possible presence of a sub-population of persistent cells within the AIEC population cannot be over looked. Persister cells, an increasingly resistant sub-population of bacteria that develop during infection, have been described for other pathogens especially those that form biofilms [62]. For AIEC persister cells may play a crucial role in maintaining a percentage of the macrophage and DC population infected and in a heightened inflammatory state. These AIEC upon their release from infected cells could act as a seeding mechanism for further infection, contributing to the persistent nature of infection as is seen in CD. In this regard rapid intracellular replication as previously reported may not be as important as the ability to persist within immune cells [13].

AIEC in the future will need to be examined as a pathogen that is not just an opportunist that can target cells defective in autophagy but as a pathogen with a distinct mechanism of infection and persistence independent of autophagy. This inhibition of apoptosis in immune cells infected with AIEC explains why AIEC as opposed to numerous other intestinal pathogens can persist so well in autophagy defective cells. An inability to remove cells through autophagy coupled with an apoptotic defect induced by AIEC infection would greatly restrict the ability of an infected cell to respond to intracellular AIEC. Understanding this dual role for PCD during AIEC infection would open up a new front in our attempts to combat AIEC induced inflammation.

## Supporting Information

Figure S1 **Generation of bone marrow derived dendritic cells (BMDCs) from C57/Bl6 mice.** BMDCs were derived using Flt3 ligand and after 7 days cells were harvested and analyzed by flow cytometry (LSR II BD Biosciences). Classical DCs (cDCs) compromised approximately 30% of cells, with 31% plasmacytoid (pDCs).
(TIF)

Figure S2 **Cytotoxicity in RAW 264.7 cells post-infection as an indicator of necrosis.** LDH activity was measured for the first 10 hpi and expressed as activity per mg of protein recovered. LDH activity assays were repeated at least three times in triplicate and data from a representative experiment is shown. Data was analyzed by an unpaired Student’s *t*-test and showed no significant difference in cytotoxicity levels between any of the infected or control samples at 3 and 6 hours, with or without 10 µM MG132.
(TIF)

Figure S3 **IL-10 release by RAW 264.7 cells over the first 72 hpi.** IL-10 release by RAW 264.7 cells was monitored over the first 72 hpi by sampling supernatants of infected RAW 264.7 cells and subjecting these to ELISA analysis. All cytokine experiments were carried out in triplicate and cytokine levels were measured in at least three independent experiments. Data was analyzed by an unpaired Student’s *t*-test. Statistically significant relationships are denoted. NS = Not significant. *P* values ***<0.005.
(TIF)

Figure S4 **IL-12/23 p40 release by RAW 264.7 cells over the first 72 hpi.** The release of the p40 subunit common to both IL-12 and IL-23 was monitored over the first 72 hpi by sampling supernatants of infected RAW 264.7 cells and testing these by ELISA. Data was analyzed by an unpaired Student’s *t*-test. Statistically significant relationships are denoted. NS = Not significant. *P* values ***<0.005.
(TIF)

Figure S5 **IL-10 release by BMDCs over the first 72 hpi.** The release of IL-10 was monitored over the first 72 hpi by sampling supernatants of infected BMDC cultures and testing these by ELISA. Data was analyzed by an unpaired Student’s *t*-test. Statistically significant relationships are denoted. NS = Not significant. *P* values *<0.01, **<0.05, ***<0.005.
(TIF)

Figure S6 **IL-12/23 p40 release by BMDCs cells over the first 72 hpi.** The release of the p40 subunit common to both IL-12 and IL-23 was monitored over the first 72 hpi by sampling supernatants of infected BMDC cultures and testing these by ELISA. Data was analyzed by an unpaired Student’s *t*-test. Statistically significant relationships are denoted. NS = Not significant. *P* values ***<0.005.
(TIF)

Figure S7 **Densitometric analysis of S-nitrosylated caspase-3 levels.** S-nitrosylated caspase-3 levels in RAW 264.7 cells (Figure 5) was subjected to densitometric analysis. Levels of S-nitrosylated pro-caspase-3 (A) ad active caspase-3 (B) in samples were compared to those of control uninfected samples which had not been treated with MG132 (10 µM). Analysis was carried out on three separate blots and a representative analysis is shown.
(TIF)
