# Supplementary figures and images for: Increased S-Nitrosylation and Proteasomal Degradation of Caspase-3 during Infection Contribute to the Persistence of Adherent Invasive Escherichia coli (AIEC) in Immune Cells
Source: PLoS One. 2013 Jul 4;8(7):e68386. doi: 10.1371/journal.pone.0068386 (PMC3701656; doi:10.1371/journal.pone.0068386)

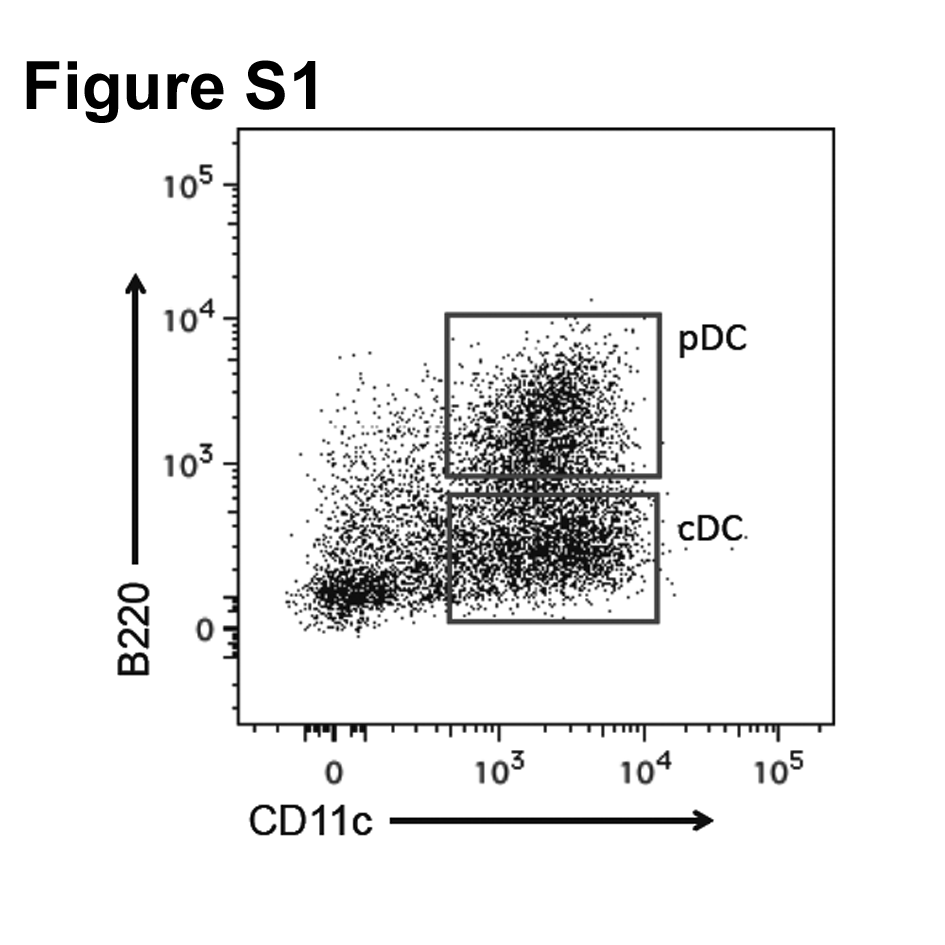

Supplement: Figure S1 — Generation of bone marrow derived dendritic cells (BMDCs) from C57/Bl6 mice. BMDCs were derived using Flt3 ligand and after 7 days cells were harvested and analyzed by flow cytometry (LSR II BD Biosciences). Classical DCs (cDCs) compromised approximately 30% of cells, with 31% plasmacytoid (pDCs). (TIF) [file pone.0068386.s001.tif]

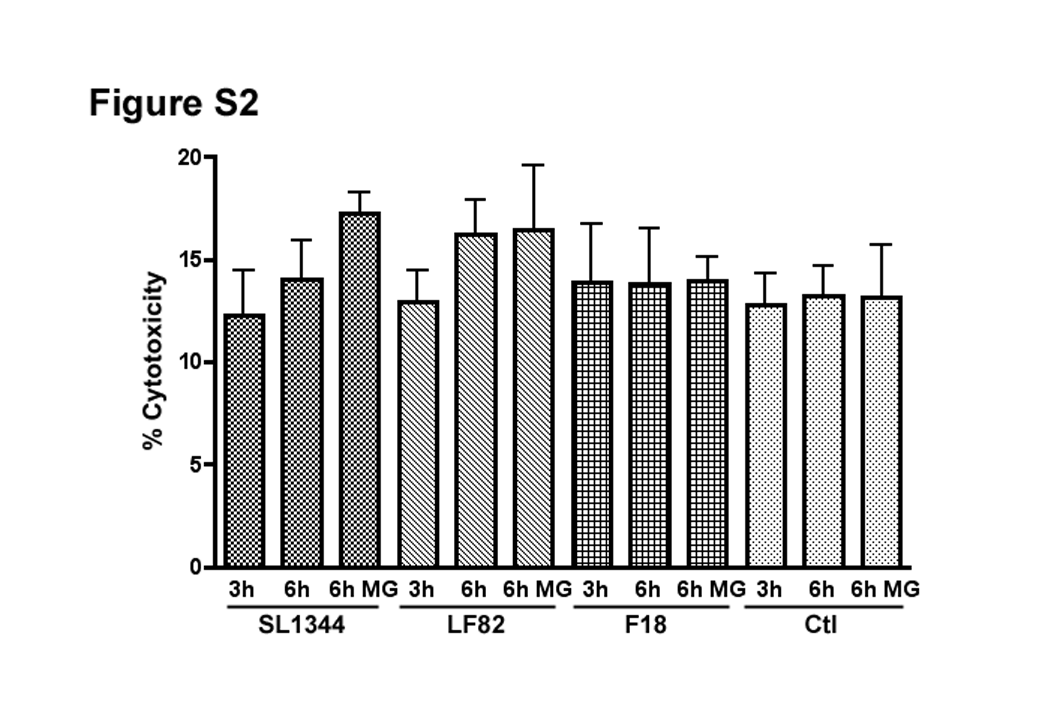

Supplement: Figure S2 — Cytotoxicity in RAW 264.7 cells post-infection as an indicator of necrosis. LDH activity was measured for the first 10 hpi and expressed as activity per mg of protein recovered. LDH activity assays were repeated at least three times in triplicate and data from a representative experiment is shown. Data was analyzed by an unpaired Student’s t-test and showed no significant difference in cytotoxicity levels between any of the infected or control samples at 3 and 6 hours, with or without 10 µM MG132. (TIF) [file pone.0068386.s002.tif]

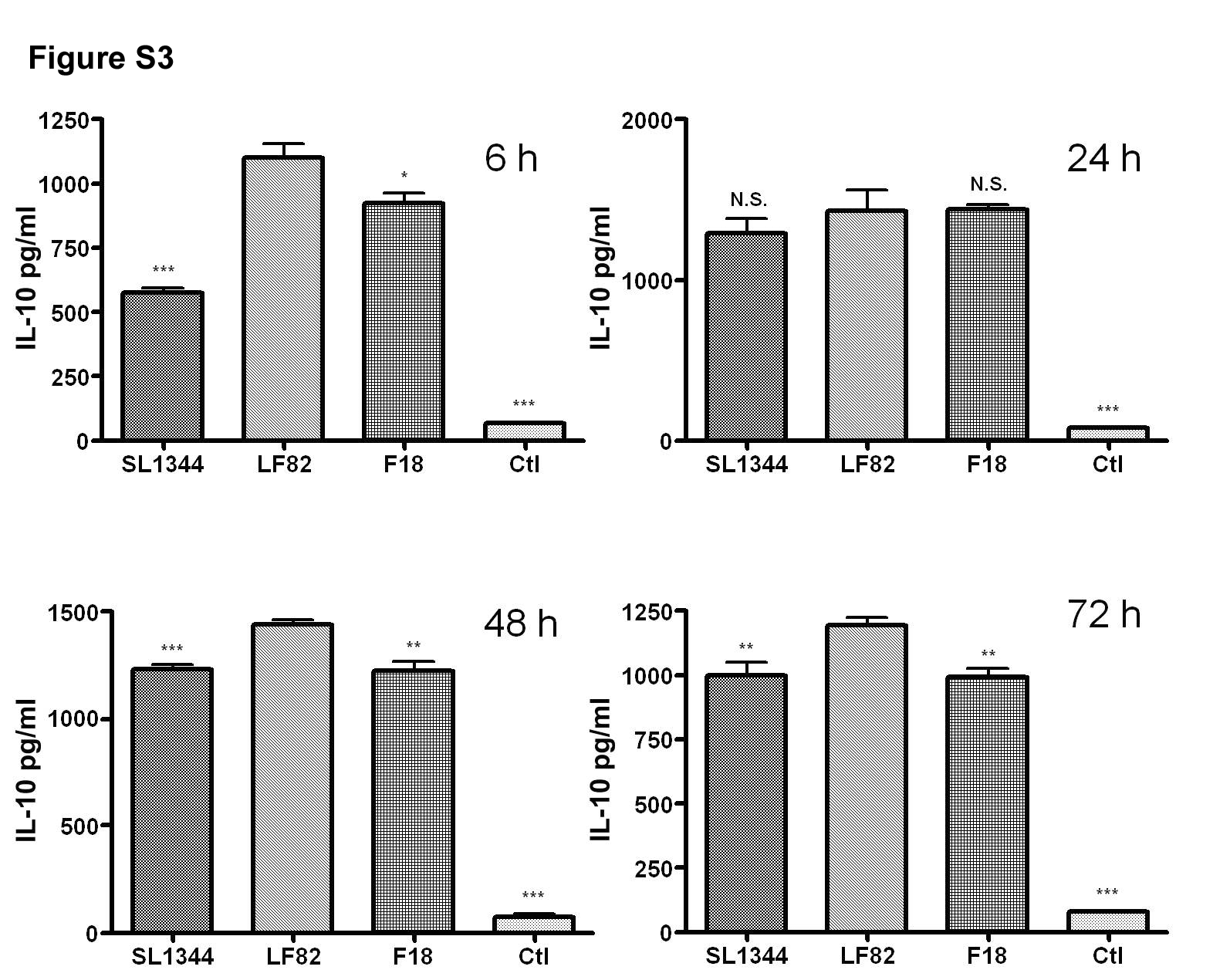

Supplement: Figure S3 — IL-10 release by RAW 264.7 cells over the first 72 hpi. IL-10 release by RAW 264.7 cells was monitored over the first 72 hpi by sampling supernatants of infected RAW 264.7 cells and subjecting these to ELISA analysis. All cytokine experiments were carried out in triplicate and cytokine levels were measured in at least three independent experiments. Data was analyzed by an unpaired Student’s t-test. Statistically significant relationships are denoted. NS = Not significant. P values ***<0.005. (TIF) [file pone.0068386.s003.tif]

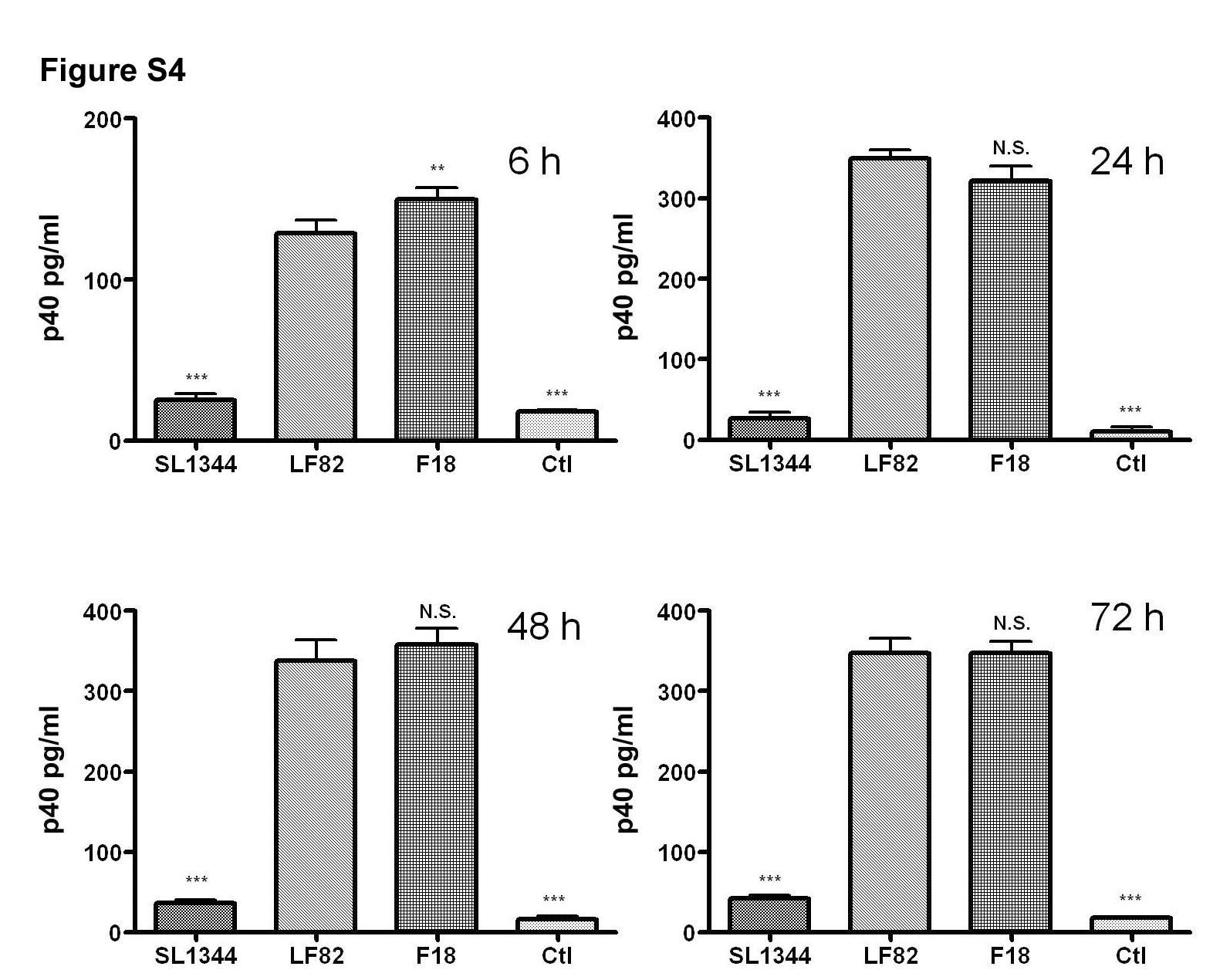

Supplement: Figure S4 — IL-12/23 p40 release by RAW 264.7 cells over the first 72 hpi. The release of the p40 subunit common to both IL-12 and IL-23 was monitored over the first 72 hpi by sampling supernatants of infected RAW 264.7 cells and testing these by ELISA. Data was analyzed by an unpaired Student’s t-test. Statistically significant relationships are denoted. NS = Not significant. P values ***<0.005. (TIF) [file pone.0068386.s004.tif]

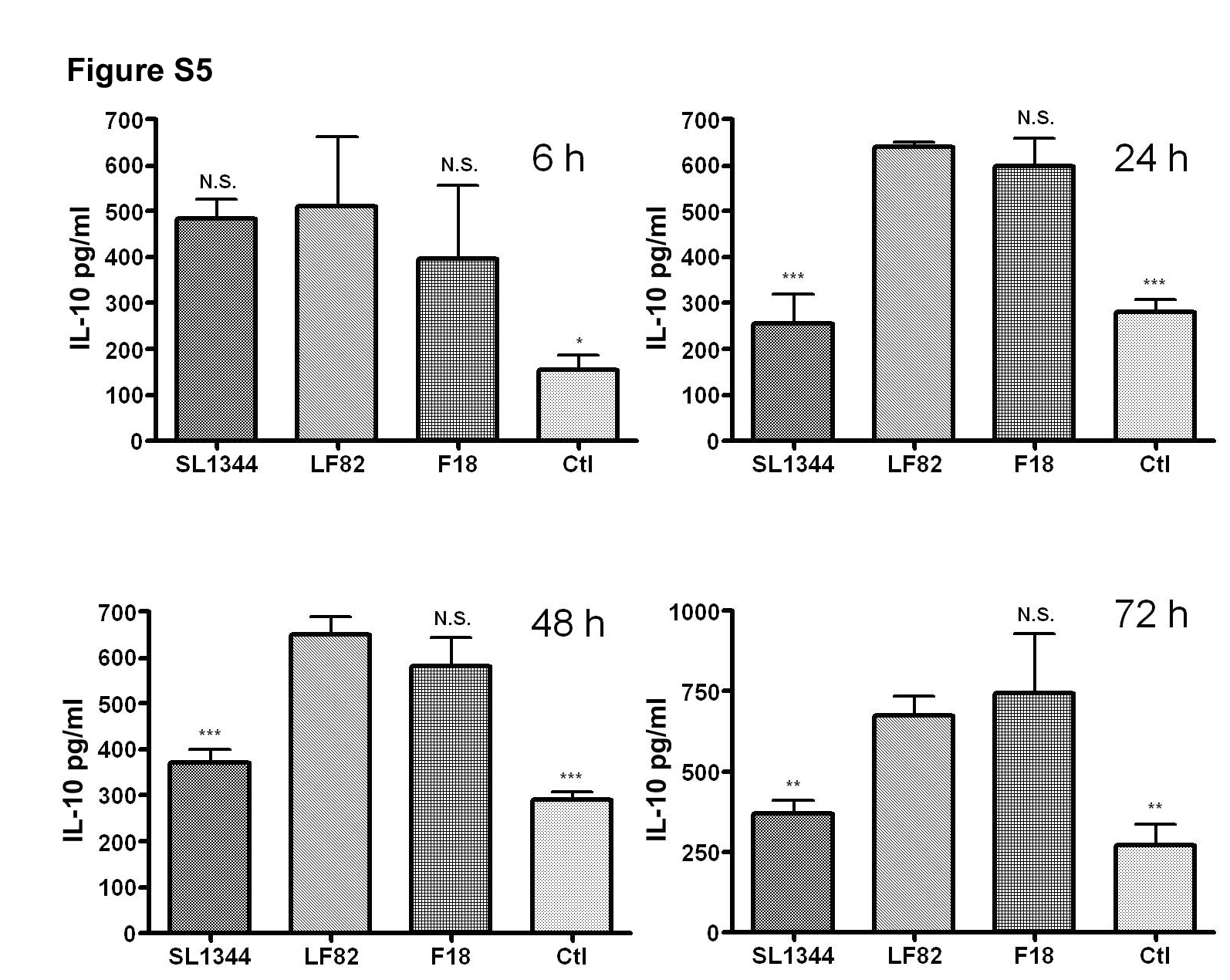

Supplement: Figure S5 — IL-10 release by BMDCs over the first 72 hpi. The release of IL-10 was monitored over the first 72 hpi by sampling supernatants of infected BMDC cultures and testing these by ELISA. Data was analyzed by an unpaired Student’s t-test. Statistically significant relationships are denoted. NS = Not significant. P values *<0.01, **<0.05, ***<0.005. (TIF) [file pone.0068386.s005.tif]

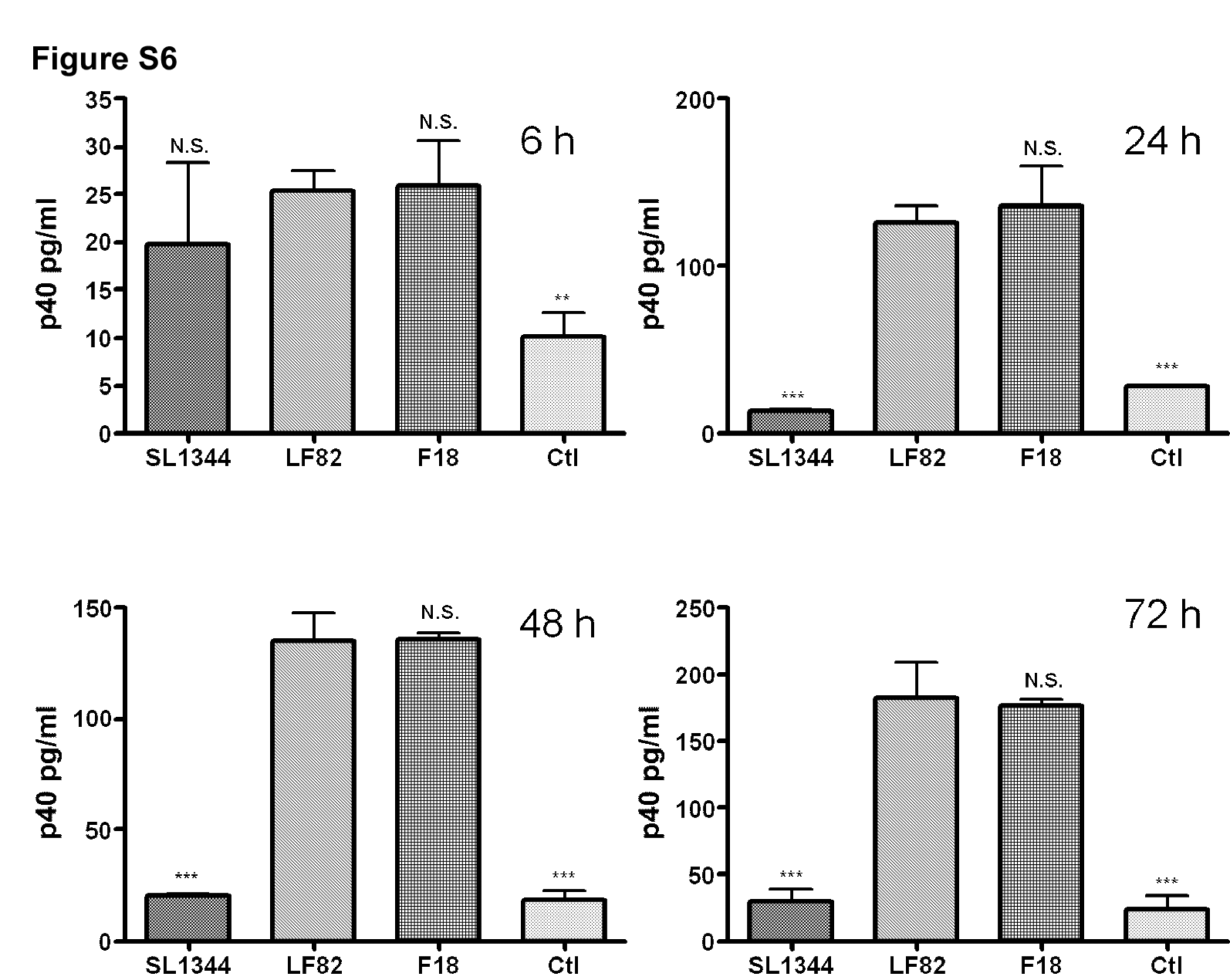

Supplement: Figure S6 — IL-12/23 p40 release by BMDCs cells over the first 72 hpi. The release of the p40 subunit common to both IL-12 and IL-23 was monitored over the first 72 hpi by sampling supernatants of infected BMDC cultures and testing these by ELISA. Data was analyzed by an unpaired Student’s t-test. Statistically significant relationships are denoted. NS = Not significant. P values ***<0.005. (TIF) [file pone.0068386.s006.tif]

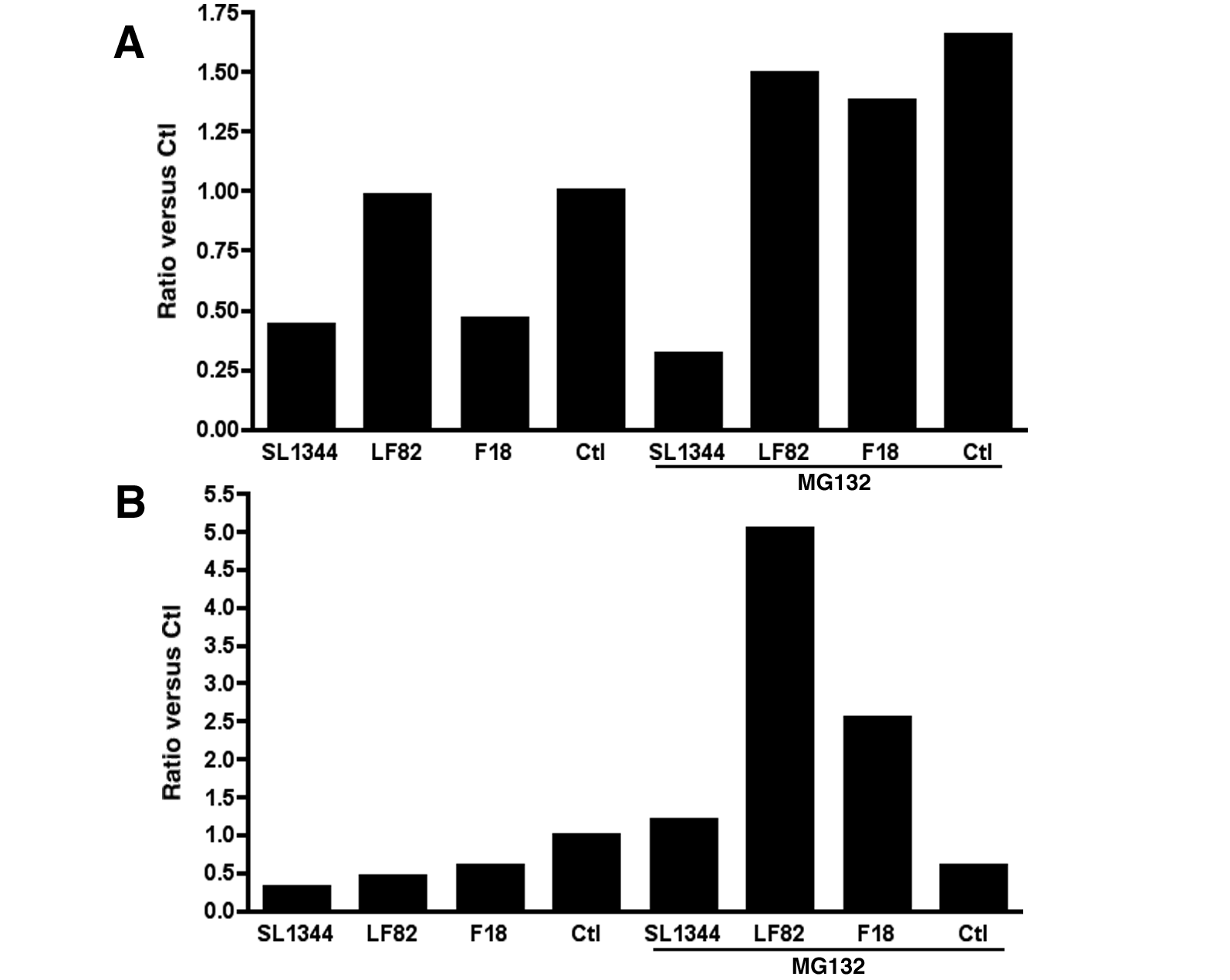

Supplement: Figure S7 — Densitometric analysis of S-nitrosylated caspase-3 levels. S-nitrosylated caspase-3 levels in RAW 264.7 cells (Figure 5) was subjected to densitometric analysis. Levels of S-nitrosylated pro-caspase-3 (A) ad active caspase-3 (B) in samples were compared to those of control uninfected samples which had not been treated with MG132 (10 µM). Analysis was carried out on three separate blots and a representative analysis is shown. (TIF) [file pone.0068386.s007.tif]
